# Supplementary material for: Role of epithelial to mesenchymal transition associated genes in mammary gland regeneration and breast tumorigenesis
Source: Nat Commun. 2017 Nov 21;8:1669. doi: 10.1038/s41467-017-01666-2 (PMC5698470; doi:10.1038/s41467-017-01666-2)
Supplement: Supplementary file 1 — Supplementary Information [file 41467_2017_1666_MOESM1_ESM.pdf]

Supplementary Figures

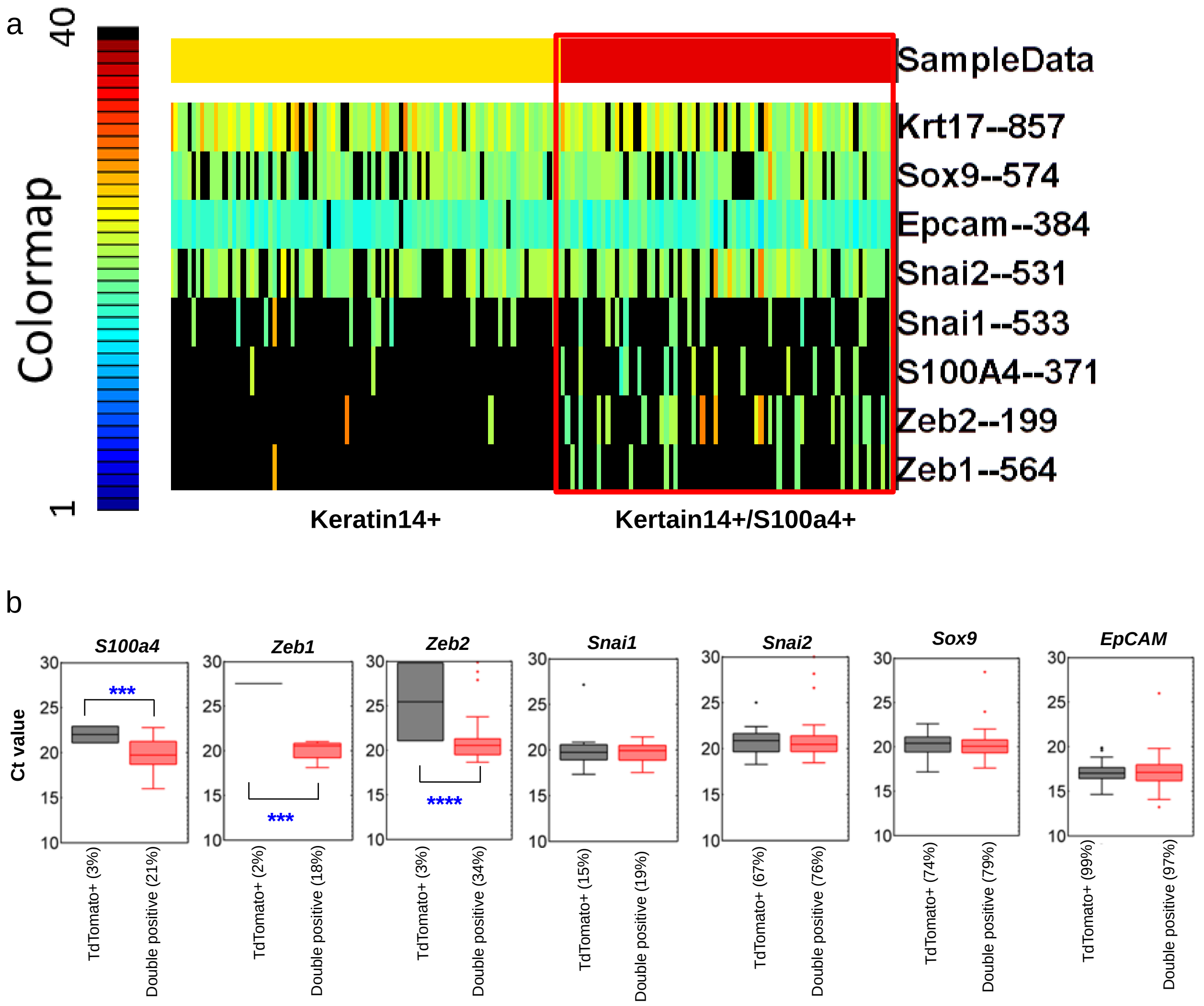

Supplementary Fig. 1: Single cell gene expression in the *K14*^{cre}*/Rosa*^{TdTomato}*/S100a4*^{GFP} mouse

(a) Raw data of single cell gene expression profiling in the *K14*^{cre}*/Rosa*^{TdTomato}*/S100a4*^{GFP} mice. Heatmap of Ct values (≤25) of selected genes in basal TdTomato+ and basal TdTomato+/GFP+ single cells (columns) and genes (rows) measured simultaneously from each cell. The percentage of positive cells is shown in Fig. 1c and median expression of each gene is shown in Supplementary Fig. 1b.

(b) Box plots for *S100a4*, *Zeb1*, *Zeb2*, *Snai1*, *Snai2*, *Sox9*, *EpCAM* and *Krt-5*. A higher Ct value indicates lower expression. Data are shown as median expression ± SD. Percentage of positive cells in each group is indicated below. * $p < 0.05$, ** $p < 0.01$, *** $p < 0.001$, **** $p<0.0001$. (Wilcoxon Ranksum Test).

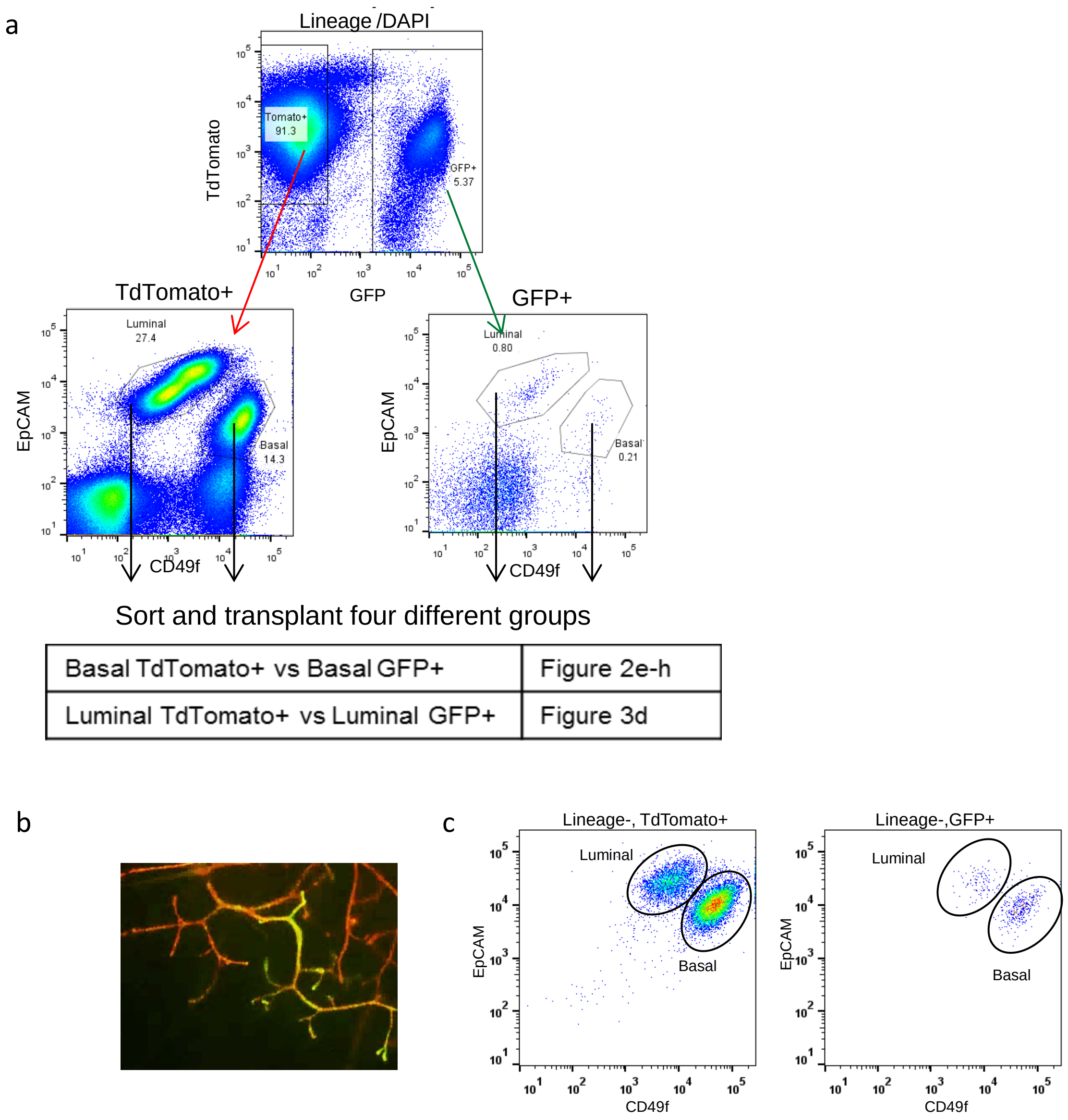

Supplementary Figure 2: Strategy for transplantation and analysis of transplant in *S100a4^{cre}/Rosa26^{mTmG}* mice

(a) Sorting schematic using *S100a4^{cre}/Rosa26^{mTmG}* mice for transplantation assays in Figure 2e and Figure 3d

(b) Representative image of TdTomato+ transplant with S100a4 switched GFP+ cells

(c) FACS analysis of basal TdTomato+ transplanted cells that contain spontaneously switched GFP+ cells. Both GFP+ and TdTomato+ transplanted cells contain luminal and basal lineages based on EpCAM and CD49f staining.

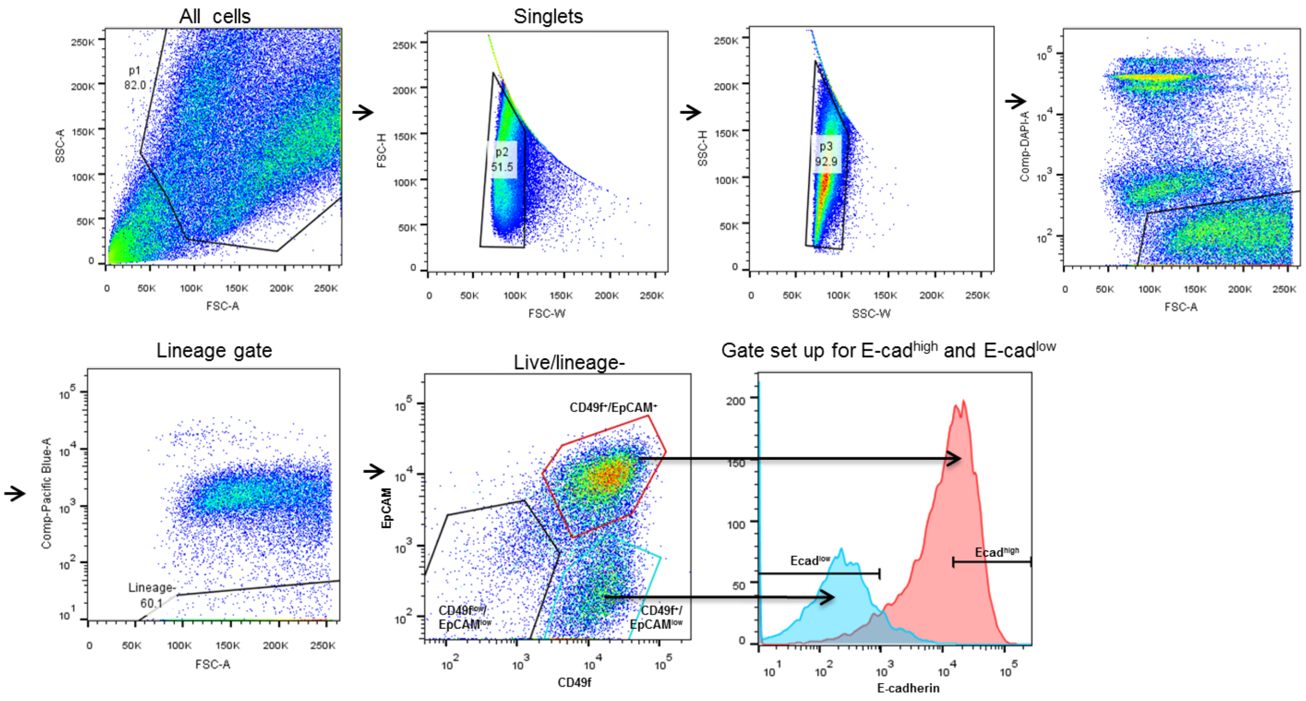

Supplementary Figure 3: Sorting gates for the PDX tumors (one representative of 5 different tumors is shown). The CD49f$^+$/EpCAM$^+$ /E-cadherin^{high} , CD49f$^+$/EpCAM$^+$ /E-cadherin^{low} gates (red histogram) are set based on expression of E-cadherin in CD49f$^+$/EpCAM^{low} population (blue histogram).

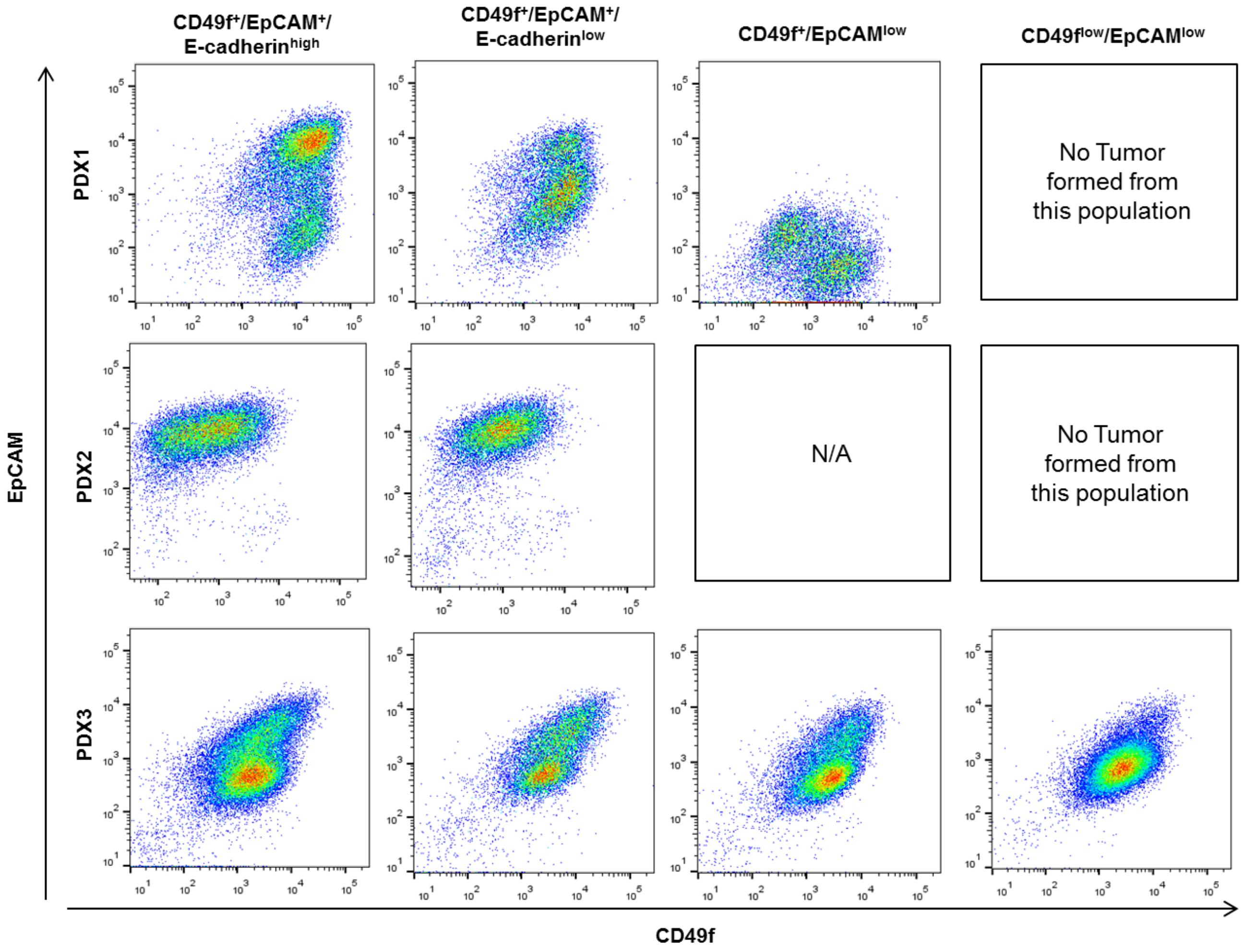

Supplementary Figure 4: Analysis of tumors derived from different populations.

(a) H2kd$^-$CD45$^-$DAPI$^-$ cells from different populations (*indicated at the top of each column*) injected in tumorigenicity assays were analyzed based on CD49f and EpCAM. FACS plots for PDX1 (upper row), PDX2 (middle row) and PDX3 (bottom row) are shown.

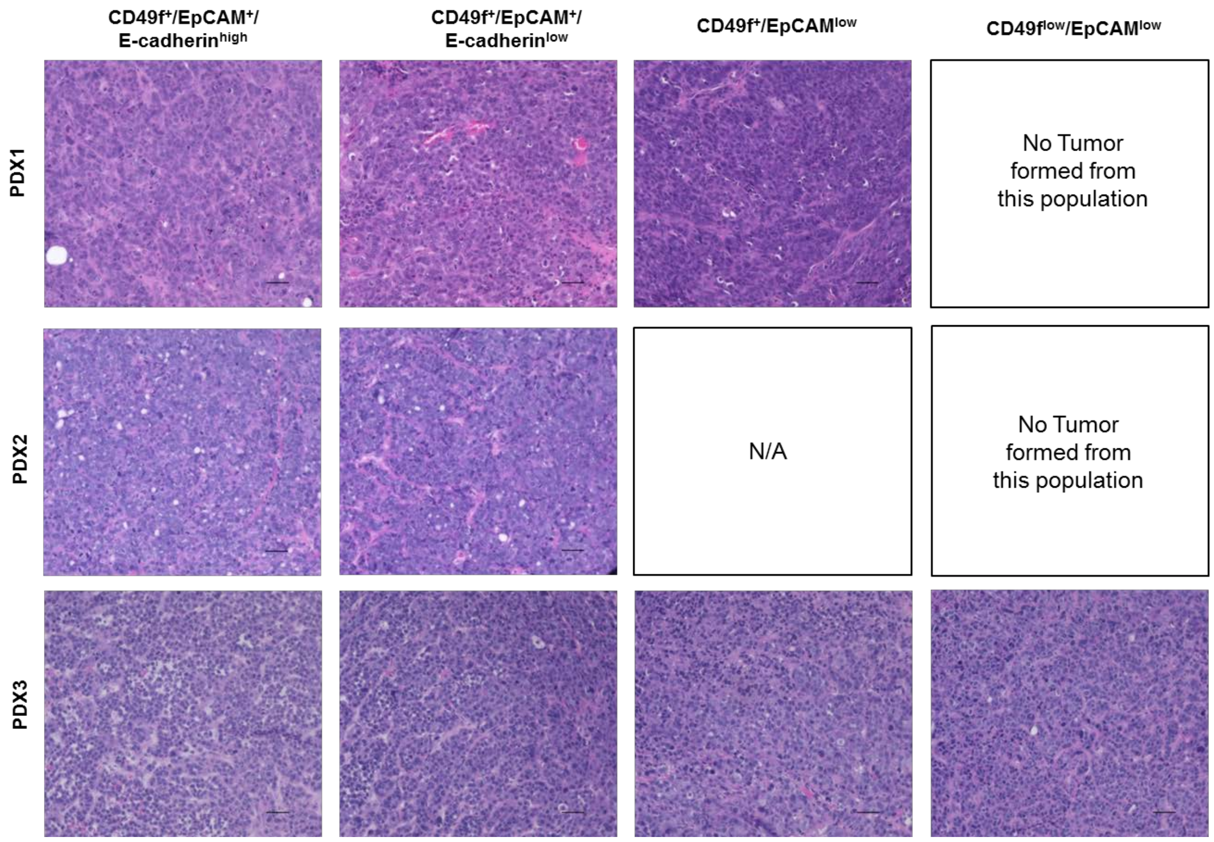

Supplementary Figure 5: Histology of tumors derived from different populations

(a) Hematoxylin and Eosin staining of tumors (*indicated at the top of each column*) formed in tumorigenicity assays. Scale bar, 50 µm

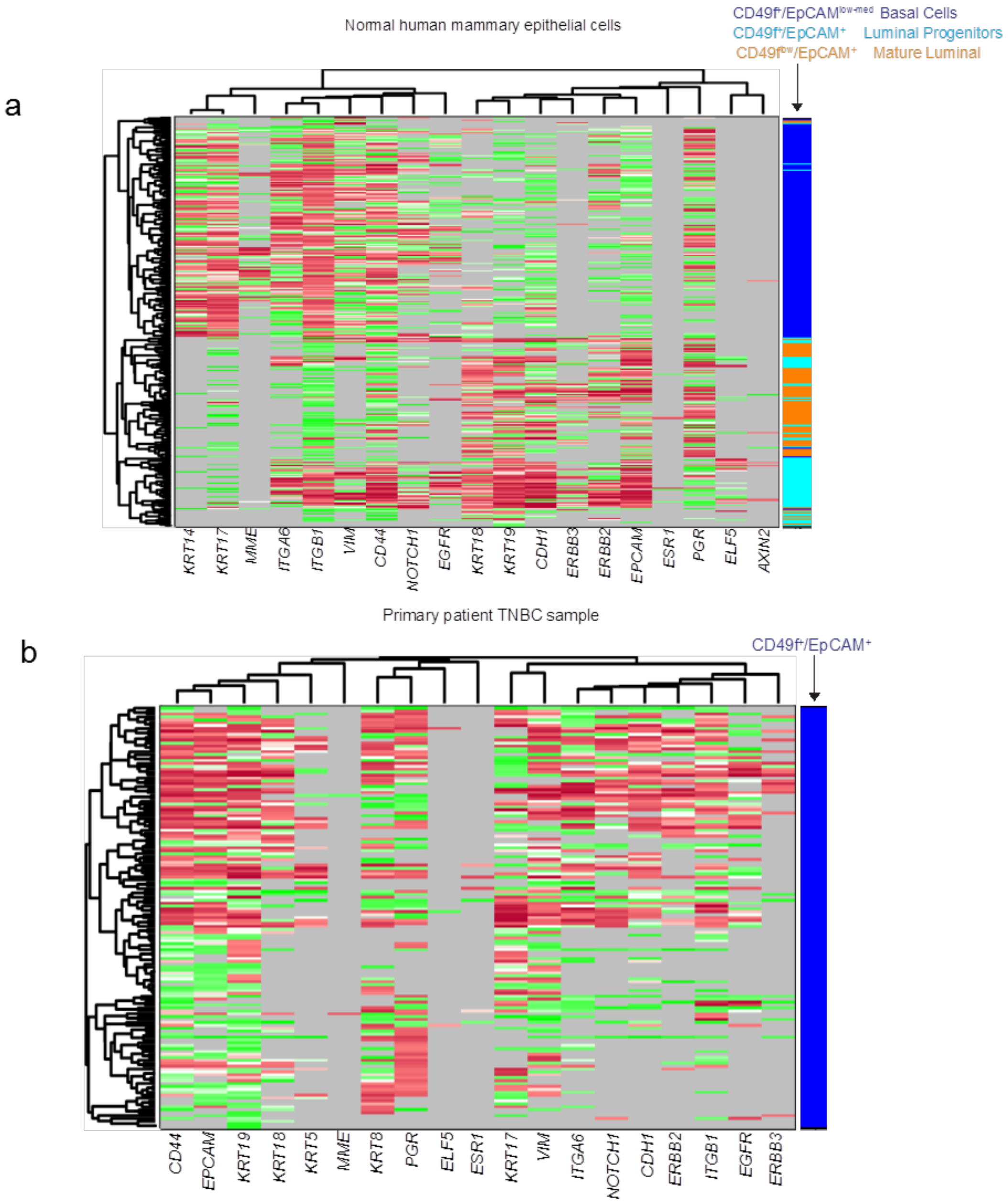

Supplementary Figure 6: Single Cell Analysis of Primary Human Samples

Gene expression heatmap of normal human primary mammary epithelial cells (a) and primary TNBC cells (b) single cells (rows) and genes (columns) measured simultaneously from each cell. Gene expression levels (in terms of threshold cycles, Ct were standardized and clustered such that phenotypically similar cells are grouped next to each other (Red – high expression, Green – low expression, Gray – no expression). Samples were obtained from the patient and directly used for single cell gene expression profiling. *Sample in b was used to generate PDX3*. Note expression of *VIM* and *KRT17* in TNBC sample across the whole population, showing that the cells do not reflect purely luminal or basal cells. CDH1^{high} cells are also CD44^{high}. Also, *ELF5*, a luminal progenitor marker is

expressed in luminal progenitors defined by $CD49f^{+}/EpCAM^{+}$ in normal mammary cells (a) is not expressed in the $CD49f^{+}/EpCAM^{+}$ in TNBC cells.

Supplementary Table 1: Description of patient derived xenografts

	Hormone status	Pathology type	Grade
PDX1	ER-/PR-/Her2-	Infiltrating ductal carcinoma	III
PDX2	ER-/PR-/Her2-	Invasive ductal carcinoma	III
PDX3	ER-/PR-/Her2-	Infiltrating ductal carcinoma	III
PDX4	ER-/PR-/Her2-	Infiltrating ductal carcinoma	III
PDX5	ER-/PR-/Her2-	Infiltrating ductal carcinoma	III

Supplementary Table 2: List of Taqman Primers

Probe id	Gene	Probe id	Gene
Mm01227384_m1	*Epcam*	Hs00153304_m1	*CD44*
Mm01306857_mH	*Krt17*	Hs00153519_m1	*MME*
Mm00503549_m1	*Krt5*	Hs00612017_s1	*PGR*
Mm00803371_m1	*S100A4*	Hs00154971_m1	*ELF5*
Mm00441531_m1	*Snai2*	Hs01046816_m1	*ESR1*
Mm03003574_s1	*Sox9*	Hs00958116_m1	*VIM*
Mm00495564_m1	*Zeb1*	Hs00413187_m1	*NOTCH1*
Mm00497199_m1	*Zeb2*	Hs99999005_mH	*ERBB2*
Mm00486918_m1	*Cdh1*	Hs00176538_m1	*ERBB3*
Mm00449201_m1	*Vim*	Hs00193306_m1	*EGFR*
Hs00243201_m1	*S100A4*	Hs00610344_m1	*AXIN2*
Hs00195591_m1	*SNAI1*		
Hs00950344_m1	*SNAI2*		
Hs00165814_m1	*SOX9*		
Hs01566407_m1	*ZEB1*		
Hs00207691_m1	*ZEB2*		
Hs01023895_m1	*CDH1*		
Hs00901885_m1	*EpCAM*		
Hs01127543_m1	*ITGB1*		
Hs01041011_m1	*ITGA6*		
Hs00361185_m1	*KRT5*		
Hs02339474_g1	*KRT8*		
Hs00559328_m1	*KRT14*		
Hs01555135_g1	*KRT17*		
Hs01920599_gH	*KRT18*		
Hs01051611_gH	*KRT19*		

Supplementary Table 3: List of Antibodies used.

Antibody	Species	Clone/Catalog #	Company	Dilution
H2kd	Mouse	SF-1.1	Biolegend	1:100
CD31	Mouse	390	Biolegend	1:100
CD45	Mouse	30-F11	Biolegend	1:100
Ter119	Mouse	Ter-119	Biolegend	1:100
CD49f	Human/Mouse	GoH3	Biolegend	1:40
EpCAM	Human	9C4	Biolegend	1:200
EpCAM	Mouse	G8.8	Biolegend	1:200
Keratin 14	Mouse	PRB-155P	Covance	1:100
Keratin 8	Mouse	Troma1	DSHB	1:100
GFP	Chicken	ab13970	Abcam	1:500
